# Proton Beam Therapy for Treatment-Naïve Hepatocellular Carcinoma and Prognostic Significance of Albumin-Bilirubin (ALBI) Grade

**DOI:** 10.3390/cancers14184445

**Published:** 2022-09-13

**Authors:** Tae Hyun Kim, Bo Hyun Kim, Joong-Won Park, Yu Ri Cho, Young-Hwan Koh, Jung Won Chun, Eun Sang Oh, Do Yeul Lee, Sung Uk Lee, Yang-Gun Suh, Sang Myung Woo, Sung Ho Moon, Sang Soo Kim, Woo Jin Lee

**Affiliations:** 1 Center for Liver and Pancreatobiliary Cancer, National Cancer Center, Goyang 10408, Korea; 2 Center for Proton Therapy, National Cancer Center, Goyang 10408, Korea

**Keywords:** hepatocellular carcinoma, overall survival, proton beam therapy, radiotherapy, albumin-bilirubin

## Abstract

**Simple Summary:**

Proton beam therapy (PBT) has not been generally recommended as an initial treatment for hepatocellular carcinoma (HCC) due to the insufficiency of data on PBT for treatment-naïve HCC until now, and albumin-bilirubin (ALBI) grade has been shown to be an effective assessment of liver dysfunction and more discriminatory for survival than the Child–Pugh classification. This study evaluated the efficacy of PBT as first-line treatment in treatment-naïve HCC and assessed the prognostic significance of ALBI grade in these patients. Our findings showed that PBT could result in comparable local tumor control and survival outcomes in treatment-naïve HCC patients to those of other recommended first-line treatments, with a safe toxicity profile compared to our institutional cohort data and previous other studies, and ALBI grade and tumor stage were independent predicting factors for overall survival.

**Abstract:**

To evaluate the efficacy of proton beam therapy (PBT) as an initial treatment in treatment-naïve hepatocellular carcinoma (HCC) patients and to assess the prognostic significance of albumin-bilirubin (ALBI) grade, 46 treatment-naïve HCC patients treated with PBT were analyzed. The ALBI grade distribution was grade 1 in 11 (23.9%) patients, grade 2 in 34 (73.9%) patients, and grade 3 in 1 (2.2%) patient. The median duration of follow-up was 56.5 months (95% confidence interval [CI], 48.2–64.7). Among the 46 patients, disease progression was observed in 23 (50%) patients: local progression in 3 (6.5%) patients; intrahepatic progression in 22 (47.8%); and extrahepatic progression in 5 (10.9%). The 5-year freedom from local progression (FFLP), progression-free survival (PFS), and overall survival (OS) rates were 92.7% (95% CI, 84.7–100.7), 43.3% (95% CI, 28.2–58.4), and 69.2% (95% CI, 54.9–83.5), respectively. In multivariate analysis, there were no independent factors for FFLP (*p* > 0.05 each), but tumor stage and ALBI grade were independent factors for PFS and OS (*p* < 0.05 each). PBT could result in comparable OS in treatment-naïve HCC patients to other recommended first-line treatments, and ALBI grade, in addition to tumor stage, could be useful for predicting OS.

## 1. Introduction

Hepatocellular carcinoma (HCC) is mostly associated with chronic liver disease related to hepatitis B and C virus (HBV and HCV) infection, alcoholic and non-alcoholic fatty liver disease, etc., and the degree of liver dysfunction, in addition to the tumor burden, is closely related to the prognosis of HCC patients [1]. Thus, the selection of the initial treatment modality for HCC is generally determined according to evidence based on tumor stage, the degree of liver dysfunction assessed by the Child–Pugh score, and performance status [2,3,4,5,6], and various local treatment modalities, including surgical resection, liver transplantation, radiofrequency ablation (RFA), percutaneous ethanol injection (PEI), transarterial chemoembolization (TACE), and transarterial radioembolization (TARE), have been applied.

Proton beam therapy (PBT) is a type of radiotherapy (RT) that has a better dose distribution due to the unique physical property of the proton beam, called the Bragg peak, than RT using X-rays. Mostly, PBT has been applied in patients with recurrent and/or residual HCC for which other local treatments are unsuitable or refused, and its local control effect and safety have been demonstrated in various previous studies [7,8,9,10,11,12,13,14,15]. Recently, a randomized controlled trial comparing PBT with RFA in patients with recurrent or residual HCC demonstrated that PBT had a comparable local control and safety profile to RFA [16]. Until now, PBT has not been generally recommended as an initial treatment for HCC due to the insufficiency of data on PBT for treatment-naïve HCC, and similar to other local treatment modalities, PBT is reserved for HCC patients with a Child–Pugh score ≤ 7. The Child–Pugh classification was originally designed to predict mortality during surgery in patients with bleeding esophageal varices [17], and is now used to assess the degree of liver dysfunction. There has been concern regarding the consistency of the Child–Pugh classification due to the subjective nature of determining the degree of ascites and severity of hepatic encephalopathy. Recently, albumin-bilirubin (ALBI) grade was shown to be an effective assessment of liver dysfunction and more discriminatory for survival than the Child–Pugh score [18]. Thus, this study was conducted to evaluate the efficacy of PBT as initial treatment in treatment-naïve HCC patients and to assess the prognostic significance of ALBI grade in treatment-naïve HCC patients treated with PBT.

## 2. Materials and Methods

### 2.1. Patients

HCC patients who received PBT between September 2012 and April 2020 were registered in a database and reviewed. The inclusion criteria for this study were as follows: (i) HCC diagnosed with histologic findings or typical radiologic findings based on guidelines from the American Association for the Study of Liver Diseases [5], the Korean Liver Cancer Study Group and the National Cancer Center (NCC) [3]; (ii) treatment naïve HCC; and (iii) age ≥ 18 years. The Barcelona Clinic Liver Cancer (BCLC) [2] and American Joint Committee on Cancer (AJCC) [19] staging classifications were used for clinical and tumor staging, respectively. The data of each patient, including age, sex, performance status, tumor size, clinical and tumor stage, baseline laboratory tests (i.e., albumin, bilirubin, α-fetoprotein [AFP], etc.), prescribed radiation dose, further treatments after PBT, times and sites of disease progression, etc., were collected from medical records. The collected data of each patient were assigned to case numbers and then anonymized. Data analyses were performed according to the relevant regulations, including the Good Clinical Practice guidelines and the Declaration of Helsinki. The present study was approved by the institutional review board of the NCC (NCC20220192), and the requirement for written informed consent was waived considering the retrospective study design.

### 2.2. Treatment

The PBT procedures have been described in detail previously [13,15,20,21]. Contrast-enhanced four-dimensional computed tomography (CT) images were obtained in each patient. The internal target volume (ITV) and organs at risk (OARs) were defined as the sum of the gross tumor volumes (GTVs) and each OAR on the CT images during the exhalation (gated) phases (i.e., 30% of the whole respiratory cycle), and the clinical target volume was considered the ITV without additional margins [13,15,20,21] (Supplementary Figure S1). The planning target volumes (PTVs) were defined as the ITV with a 5–7 mm margin in all directions. The PBT plan (Eclipse, Version 13.7 Varian Medical System, Palo Alto, CA, USA) was conducted using 2–4 (median, 3) 230 MeV double-scattered proton beams (Proteus 235; Ion Beam Applications, S.A., Louvain-la-Neuve, Belgium) with the intention of covering at least 95% of the PTV with 100% of the prescribed dose. The radiation doses for PBT in each patient were expressed in Gray equivalent (GyE = physical dose of proton beam [Gray] × relative biologic effectiveness of proton beam [1.1]) and the equivalent dose in a 2 Gy fraction (EQD2 [GyE_3_ or GyE_10_] = [(fraction dose + α/β)/(2 + α/β)] × total dose, with α/β values of 3 (late effects) and 10 (acute effects and tumor)) [22]. The dose-volume constraints for the OARs have been described in detail [13,15,20,21]. In brief, the relative volumes of the total liver and remaining residual liver (total liver—GTV) receiving more than 27 GyE were less than 50% and 60%, respectively; the maximum dose to the spinal cord was less than 39 GyE_3_; and the absolute volumes of the duodenum and small and large bowel received more than 35 GyE, the stomach receiving more than 37 GyE, and the esophagus receiving more than 39 GyE were less than 2 cm^3^. Five dose-fractionation regimens were used for PBT depending on tumor location and proximity of gastrointestinal (GI) organs: for the GI protocol, a total of 50 or 60 GyE in 10 fractions (EQD2, 62.5 GyE_10_ or 80 GyE_10_) were administered for patients with tumors located less than 1 cm and 1-2 cm from GI organs, respectively; for the standard protocol, 66 GyE was administered in 10 fractions (EQD2, 91.3 GyE_10_) for patients with tumors located more than 2 cm from GI organs; and for the dose-escalation protocol, 70 GyE was administered in 10 fractions (EQD2, 99.2 GyE_10_) or 52.8 GyE in 4 fractions (EQD2, 102.1 GyE_10_) for patients with tumors located more than 2 cm from GI organs. For each treatment, all patients were asked to fast for at least 4 h prior to treatment, and after verifying each patient’s position and isocenter using digital orthogonal and/or cone beam CT images, radiation was delivered during gated phases with a respiratory-gated technique.

### 2.3. Assessments and Statistical Analysis

Clinical assessments, laboratory tests including AFP, and radiological examinations including dynamic liver CT and/or magnetic resonance imaging (MRI) were performed in the first month, every 3 months for the following 2 years, and every 6 months thereafter. Vascular invasion of the main branches of the portal vein (main, right or left portal vein), three hepatic veins (right, middle, or left), or the hepatic artery (proper, right or left) was defined as major vascular invasion, and vascular invasion of sectional and segmental branches was defined as non-major vascular invasion. The albumin-bilirubin (ALBI) score was calculated from the formula ALBI score = [log10 (bilirubin (μmol/L)) × 0.66] + [albumin (g/L)] × (−0.085)], and the ALBI grades were classified by specific cutoff values: grade 1, ≤−2.60; grade 2, −2.60< and ≥−1.39; and grade 3, >−1.39 [18]. The tumor assessments, including size and response, and the evaluation of disease progression were performed according to the Response Evaluation Criteria in Solid Tumors (RECIST) version 1.1 [23]. Disease progression was classified according to its sites, as follows: local progression was defined as the presence of a growth or new tumor within 1 cm from the margin of the PTV; intrahepatic progression was defined as the presence of growth or a new tumor within the liver, except for local progression; and extrahepatic progression was defined as growth or a new tumor outside of the liver, such as regional or non-regional lymph nodes and distant organs (Supplementary Figure S1). The Common Terminology Criteria for Adverse Events (version 4.03) were used for the assessment of PBT-related adverse events (AEs). The overall survival (OS), progression-free survival (PFS), and freedom from local progression (FFLP) times were determined from the commencement date of PBT to the date of death from any cause or the last follow-up, any disease progression or death, and local progression, respectively. Fisher’s exact test was used to compare the distributions of categorical variables, and correlations among variables were assessed using Spearman’s rank correlation coefficient test. The Kaplan–Meier method was used to estimate the probability of survival, and in univariate analysis, the survival differences were compared among variables using the log-rank test. Multivariate analysis using a stepwise forward selection procedure was performed with the variables with *p* < 0.1 in the univariate analysis, and a Cox proportional hazards model was used to estimate the hazard ratios (HRs). All statistical analyses were performed using STATA software version 14.0 (StataCorp, College Station, TX, USA), and a *p* value < 0.05 indicated statistical significance.

## 3. Results

Between September 2012 and April 2020, 487 patients received PBT at our institute. Among them, 46 treatment-naïve HCC patients, for whom other local treatments were unsuitable or refused, received PBT according to the practice guidelines [2,3,4,5] and were included in this study. Patient characteristics are shown in Table 1.

The median age was 62 years (range, 44–81 years). The Child–Pugh score distribution was as follows: score 5, 41 (89.1%) patients; score 6, four (8.7%) patients; and score 7, one (2.2%) patient. The median ALBI score was -2.36 (range, −3.14–−1.17), and the ALBI grade distribution was as follows: grade 1, 11 (23.9%) patients; grade 2, 34 (73.9%) patients; and grade 3, one (2.2%) patient. Most (n = 40, 87%) patients had HCC with no vascular invasion, and the remaining six (13%) patients had HCC with vascular invasion: one (2.2%) patient had non-major vascular invasion, and ifve (10.8%) patients had major vascular invasion. The median EQD2 of PBT was 95.8 GyE_10_ (range, 62.5–102.1): 39 (84.8%) patients received >90 GyE_10_; and seven (15.2%) patients received ≤90 GyE_10_. After PBT, all patients with major vascular invasion received subsequent systemic treatments with sorafenib (n = 4) or doxorubicin plus cisplatin (n = 1). The median duration of follow-up for all patients was 56.5 months (95% confidence interval [CI], 48.2–64.7 months; range, 2.7–115.2 months).

**Figure 1:**
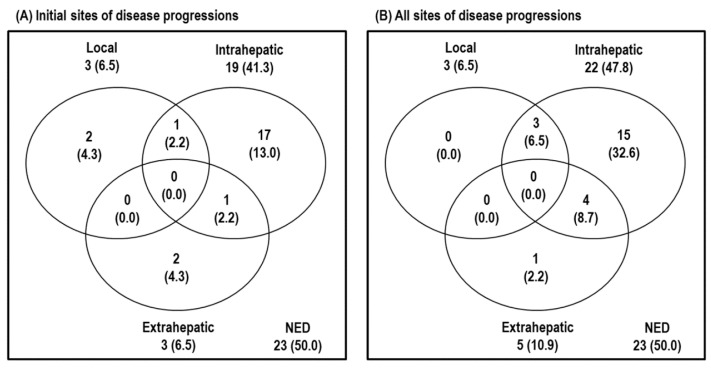
Patterns of disease progression at the time of analysis. (**A**) Initial sites of disease progression and (**B**) all sites of disease progression. Abbreviations: NED, no evidence of disease progression.

Of the 46 patients, 31 patients are still alive, and 15 patients died from disease progression (n = 11), underlying chronic renal failure (n = 2), and unknown causes (n = 2). Disease progression developed in 23 of 46 (50%) patients. The initial sites of disease progression were local sites in three (6.5%) patients, intrahepatic sites in 19 (41.3%) patients, and extrahepatic sites in three (6.5%) patients, and all of the sites of disease progression were local sites in three (6.5%) patients, intrahepatic sites in 22 (47.8%) patients, and extrahepatic sites in five (10.9%) patients (Figure 1).

The median times to local, intrahepatic and extrahepatic disease progression were 13.3 months (range, 5.3–23.9), 18.0 months (range, 0.3–83.4) and 4.8 months (range, 0.9–32.8), respectively. After confirming disease progression, all patients received subsequent treatments, such as one or combinations of local and/or systemic treatments (i.e., radiofrequency ablation (RFA), TACE, PBT, RT, sorafenib, lenvatinib, etc.) (Supplementary Table S1).

The median FFLP time was not reached, and the 3-year and 5-year FFLP rates were 92.7% (95% CI, 84.7–100.7) and 92.7% (95% CI, 84.7–100.7), respectively (Supplementary Figure S2A). In univariate analysis, patients with vascular invasion into the main and first branches and AJCC stage III/IV disease had significantly lower FFLP rates than those with no vascular invasion, vascular invasion into the segmental branches, and AJCC stage I/II disease (*p* < 0.05 each) (Table 2) (Supplementary Figure S3A,B).

Patients who received ≤90 GyE_10_ had a trend toward lower FFLP rates than those who received >90 GyE_10_, but this difference was not significant (83.3% vs. 94.4%, *p >* 0.05) (Table 2) (Supplementary Figure S3C). Not surprisingly, most (5/6, 83.3%) patients with AJCC stage III/IV disease had major vascular invasion. In addition, anatomically, major vessels of the liver, including the portal vein, are close to GI organs, including the stomach and duodenum. The proportion of patients who had major vascular invasion who received an EQD2 ≤ 90 GyE_10_ was higher than that of patients who had no vascular invasion or non-major vascular invasion (4/5 (80%) vs. 3/41 (7.3%), *p* < 0.001). Thus, EQD2 was negatively correlated with vascular invasion (*r* = −0.630, *p* < 0.001) and AJCC stage (*r* = −0.734, *p* < 0.001). However, there were no significant independent pretreatment factors associated with FFLP in the multivariate analysis because of the small number of study participants (n = 46) (*p* > 0.05 each) (Table 3).

**Figure 2:**
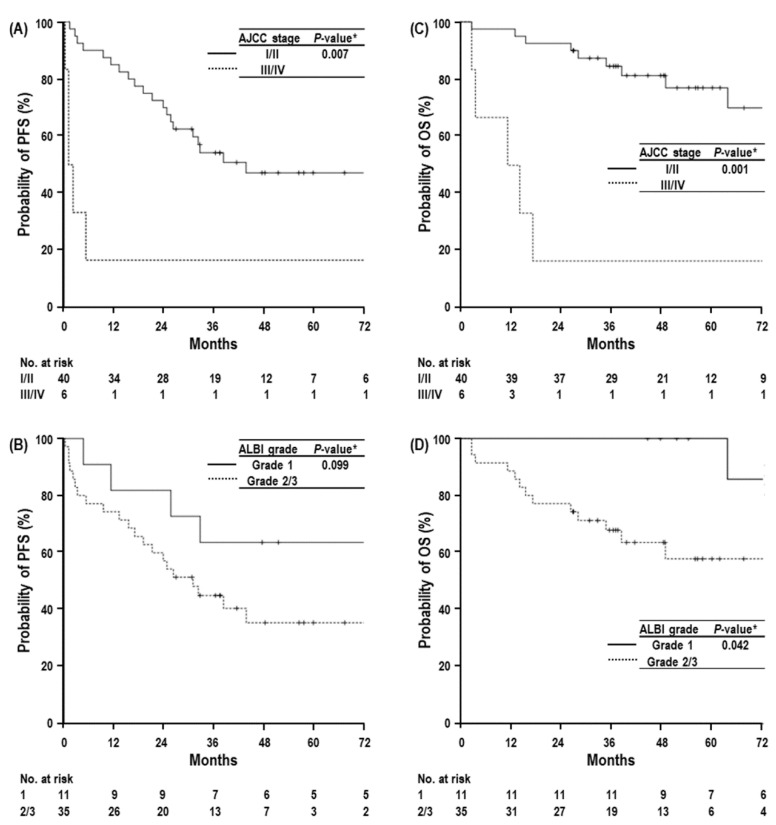
Progression-free survival (PFS) curves according to AJCC stage (**A**) and ALBI grade (**B**) in all patients, and overall survival (OS) curves according to AJCC stage (**C**) and ALBI grade (**D**). Abbreviations: AJCC stage, American Joint Committee on Cancer stage; ALBI grade, albumin-bilirubin grade. * log-rank test.

The median PFS time was 32.7 months (95% CI, 46.8–161.8), and the 3-year and 5-year PFS rates were 49.3% (95% CI, 34.5–63.9) and 43.3% (95% CI, 28.2–58.4), respectively (Supplementary Figure S2B). In univariate analysis, patients with major vascular invasion, AJCC stage III/IV disease, and EQD2 ≤90 GyE_10_ had significantly lower PFS rates than those with no vascular invasion or non-major vascular invasion, AJCC stage I/II disease, and EQD2 > 90 GyE_10_ (*p* < 0.05 each) (Table 2) (Figure 2A), and patients with a tumor size ≥ 3 cm, BCLC stage of C, and ALBI grade of 2/3 had a trend toward lower PFS rates than those with a tumor size < 3 cm, BCLC stage of 0/A/B, and ALBI grade of 1 (*p* *>* 0.05 each) (Table 2) (Figure 2B). In multivariate analysis, ALBI grade and AJCC stage were significantly associated with PFS (*p* < 0.05 each) (Table 3).

The median OS time was 104.3 months (95% CI, 46.8–161.8), and the 3-year and 5-year OS rates were 75.7% (95% CI, 63.2–88.2) and 69.2% (95% CI, 54.9–83.5), respectively (Supplementary Figure S2C). Similar to PFS, tumor size, vascular invasion, AJCC stage, BCLC stage, ALBI grade, and EQD2 were significantly associated with OS in univariate analysis (*p* < 0.05 each) (Table 2) (Figure 2C,D).

Vascular invasion was correlated with tumor size (*r* = 0.398, *p* = 0.006), AJCC stage (*r* = 0.902, *p* < 0.001), and BCLC stage (*r* = 0.824, *p* < 0.001) but was not correlated with ALBI grade (*r* = 0.032, *p* = 0.833). The median OS times and 5-year OS rates followed similar trends regarding vascular invasion, BCLC stage and AJCC stage: 104.3 months (95% CI, Not Available (NA)) vs. 14.1 months (95% CI, 0.0–36.8) and 75.1% (60.6–89.6) vs. 20.0% (−15.1–55.1) for vascular invasion (no or non-major vs. major): 104.3 months (95% CI, NA) vs. 14.1 months (95% CI, 6.7–21.5) and 76.7% (95% CI, 58.1–95.3) vs. 28.6% (−4.9–62.1) for BCLC stage (0/A/B vs. C); and 104.3 months (95% CI, NA) vs. 11.2 months (95% CI, 0.0–23.9) and 77.0% (95% CI, 62.5–91.5) vs. 16.7% (−13.1–46.5) for AJCC stage (I/II vs. III/IV) (Table 2). In multivariate analysis, AJCC stage and ALBI grade were independent factors for OS (*p* < 0.05 each) (Table 3).

The AEs related to PBT are summarized in Table 4.

Among the 46 patients, grade 1 elevated alanine aminotransferase, hyperbilirubinemia, and hypoalbuminemia without evidence of disease progression were observed in 10 (21.7%), 1 (2.2%), and 1 (2.2%) patients, respectively, and an increased Child–Pugh score was observed in one (2.2%) patient (1-point decrease). Grade 1 and 2 leukopenia was observed in five (10.9%) and two (4.3%) patients, respectively, and grade 1 thrombocytopenia was observed in seven (15.2%) patients. Grade 1 and 2 dermatitis was observed in 11 (33.9%) and two (4.3%) patients, respectively, and grade 1 radiation pneumonitis (asymptomatic and radiographic change) was observed in seven (15.2%) patients. PBT-related ≥grade 3 AEs, hepatic failure, and death were not observed. The incidences of hematologic and non-hematologic AEs were not significantly different according to ALBI grade (*p* > 0.05 each) (Table 4). The change in Child–Pugh score was not significantly related to ALBI grade and tumor location (*p* > 0.05 each) (Supplementary Table S2). The change in ALBI score was 0.13 ± 0.11 (5.0 ± 4.0%), and increased ALBI grade was observed in four (8.7%) patients. Tumor location was not significantly related with change of ALBI grade (*p* > 0.05 each) (Supplementary Table S2). The change in ALBI grade (0 vs. +1) was not significantly related with OS (5-year, 65.8% [95% CI, 49.9–81.7] vs. 100% [95% CI, NA], respectively) (*p* = 0.101).

## 4. Discussion

In patients with treatment-naïve HCC, the recommended first-line treatments are surgical resection, ablative treatments including RFA, and liver transplantation for BCLC 0 or A patients and TACE for BCLC B patients, and the reported 5-year OS rates are 70–90% for BCLC 0 patients, 50–70% for BCLC A patients, and 25–40% for BCLC B patients [2,3,4,5,6,24]. Similarly, in our institutional cohort data, patients treated with surgical resection, ablative treatments and liver transplantation demonstrated 5-year OS rates of 72.4–84.0% for BCLC 0 patients, 61.2–69.5% for BCLC A patients, and 17.7–43.0% for BCLC B patients [25]. In the present study, although PBT was applied in patients with treatment-naïve HCC for whom other local treatments were unsuitable or refused, this treatment resulted in a 5-year FFLP rate of 94.5% and a 5-year OS rate of 76.7% for BCLC 0/A/B patients (mainly A (92.3%)); these data are similar to our previous reports of PBT for patients with recurrent or residual HCC [13,14]. Fukuda et al. [9] analyzed treatment-naïve HCC patients treated with PBT and reported 5-year FFLP and OS rates of 94% and 69% for BCLC 0/A patients (n = 30), respectively, and 5-year FFLP and OS rates of 87% and 66%, respectively, for BCLC B patients (n = 34). In a recent randomized phase III trial, PBT showed noninferior 4-year FFLP rates (85.8% vs. 77.6%, p = 0.114) and similar 4-year OS rates (74% vs. 78%, p = 0.600) to RFA in patients with recurrent and/or residual HCC [16]. These findings suggested that PBT could result in comparable local tumor control and survival outcomes in treatment-naïve BCLC 0/A/B patients with HCC and was comparable to other local treatment modalities, including surgical resection, ablative treatments, and TACE.

In BCLC C patients, major vascular invasion is frequent negative prognostic factor due to rapid intra- and extrahepatic tumor spread, deterioration of liver function by impairment of blood flow of the liver, and limited treatment options [26,27,28]. Although systemic treatments are considered first-line treatments [2,3,4,5,6,29,30,31,32,33], systemic treatments have modest survival benefits (approximately 2–3 months) and low objective response rates (less than 30%) [2,3,4,5,6,29,30,31,32,33]. Thus, local treatments, such as TACE and/or RT, including PBT, have been attempted for BCLC C patients to reduce lesions with vascular invasion, including tumor thrombosis, and facilitate subsequent treatments [20,21,24,34,35]. A randomized controlled trial comparing TACE plus RT with sorafenib showed that TACE plus RT had a significantly higher radiologic response rate (33.3% vs. 2.2%), longer median time to progression (31.0 weeks vs. 11.7 weeks) and longer OS time (55 weeks vs. 43.0 weeks) than sorafenib (*p* < 0.05 each). However, when applying RT, including PBT, careful consideration is needed to avoid GI AEs due to the proximity of GI organs to major vessels of the liver, including the portal vein. In the present study, although most (71.4%) BCLC C patients had major vascular invasion, they received PBT with ≤90 GyE_10_ to minimize the risk of GI AEs, and PBT showed good local tumor control and survival, i.e., a 5-year FFLP rate of 80%, a median OS time of 14.1 months and a 5-year OS rate of 28.6%, without grade 3 AEs (0%). Even with the relatively small number of BCLC C patients with HCC (n = 7), these findings suggest that the addition of PBT to systemic treatments might be beneficial in BCLC C patients. Further large-scale comparative studies are warranted to evaluate the benefit of PBT in addition to systemic treatments in advanced HCC patients.

The degree of liver dysfunction and tumor stage are important factors for predicting OS in HCC patients [1]; the ALBI score was recently developed as an objective measure that uses serum concentrations of albumin and bilirubin to assess the degree of hepatic dysfunction and has been validated in patients with different stages of HCC treated with various local and systemic treatment modalities, including surgical resection, RFA, TACE, and sorafenib [18,36]. In HCC patients treated with stereotactic body RT, baseline ALBI grade has also been reported as a predictor for toxicity and OS [37,38,39,40]. In the present study, ALBI grade and AJCC stage were independent factors for OS in multivariate analysis (Table 3 and Table 4). These findings suggested that ALBI grade could be a more discriminating factor for OS in HCC patients treated with PBT than Child–Pugh score. However, in the present study, the predictive ability of ALBI grade and change of ALBI grade for PBT-related toxicity was not thoroughly evaluated because of the low incidence of PBT-related AEs and the relatively small number of study population (n = 46). In addition, impacts of ALBI grade and changes of ALBI grade on treatment selection for progressive diseases and OS and probable prognostic factors, including the patterns of failures after PBT and/or subsequent treatment after disease progressions, were also not thoroughly assessed. Thus, further large-scale studies are warranted to validate these findings.

The present study has several limitations. First, the potential selection bias resulting from the retrospective nature of the present study was not thoroughly assessed due to heterogeneity (i.e., different dose-fractionation schemes, details of post-PBT treatment modalities, etc.) and the relatively small number (n = 46) of study participants. To exclude the impacts of previous treatment modalities and previously treated HCC lesions on prognosis and degree of liver dysfunction, the present study included only treatment-naïve HCC patients to evaluate the efficacy of PBT as first-line treatment in these patients. Second, AEs may be underestimated in retrospective studies because of recall bias and incompleteness in medical records. Similar to the present study, several prospective studies of previously treated or untreated patients with HCC of various stages also showed a favorable safety profile, with a grade 3 AE rate ≤ 5% [7,8,11,12,14,15,16].

## 5. Conclusions

The present study showed that PBT can result in comparable OS in treatment-naïve HCC patients to other recommended first-line treatments [2,3,4,5,6,24], and ALBI grade, as an objective factor for assessing the degree of liver dysfunction, can be useful for predicting OS in HCC patients treated with PBT. Further prospective large-scale studies comparing PBT in treatment-naïve HCC patients with other local treatment modalities are needed to verify these findings.

## Figures and Tables

**Table 1:** Patient and treatment characteristics.

Characteristics		n (%)
Sex	Male	35 (76.1)
	Female	11 (23.9)
Age, years	Median (range)	62 (44–81)
	<65	27 (58.7)
	≥65	19 (41.3)
ECOG PS	0	44 (95.7)
	1	2 (4.3)
Etiology of LC	HBV	29 (63.0)
	HCV	6 (13.1)
	Alcoholic	4 (8.7)
	Unknown	7 (15.2)
Child–Pugh score	5	41 (89.1)
	6	4 (8.7)
	7	1 (2.2)
AFP, ng/mL	Median (range)	10.8 (1.3–294,089.0)
	<10	22 (47.8)
	≥10	24 (52.2)
Tumor size, cm	Median (range)	2.6 (1.0–16.0)
	≤3	26 (56.5)
	>3	20 (43.5)
Tumor location	Hilar	19 (41.3)
	Non-hilar	27 (58.7)
Vascular invasion	No	40 (87.0)
	Non-major	1 (2.2)
	Major	5 (10.8)
AJCC stage	I	37 (80.4)
	II	3 (6.5)
	III	5 (10.9)
	IV	1 (2.2)
BCLC stage	0	1 (2.2)
	A	36 (78.3)
	B	2 (4,3)
	C	7 (15.2)
Albumin (g/dL)	Median (range)	42.2 (31–51)
Total bilirubin (μmol/L)	Median (range)	70.0 (35.4–168.0)
ALBI score	Median (range)	−2.36 (−3.14–−1.17)
ALBI grade	1 (≤−2.60)	11 (23.9)
	2 (−2.60< and ≥−1.39)	34 (73.9)
	3 (>−1.39)	1 (2.2)
Post-Tx	No	41 (89.1)
	Yes	5 (10.9)
	Sorafenib	4 (8.7)
	AP	1 (2.2)
Total dose (EQD2, GyE_10_)	Median (range)	95.8 (62.5–102.1)
	≤90	7 (15.2)
	>90	39 (84.8)
Planning target volume, cm^3^	Median (range)	20.8 (8.0–627.0)
Total liver (TL) volume, mL	Median (range)	1177.3 (645.8–3211.0)
_TL_V_27GyE_, %	Median (range)	12.5 (3.8–65.3)
Remaining residual liver (RRL) volume, cm^3^	Median (range)	1049.4 (593.9–2032.4)
_RRL_V_27GyE_, %	Median (range)	10.2 (3.0–36.8)
_Stomach_D_2cc_, GyE	Median (range)	0.0 (0.0–34.0)
_Esophagus_D_2cc_, GyE	Median (range)	0.0 (0.0–39.1)
_Duodenum_D_2cc_, GyE	Median (range)	0.0 (0.0–34.4)
_Bowel_D_2cc_, GyE	Median (range)	0.0 (0.0–17.5)
_Cord_D_2cc_, GyE	Median (range)	0.0 (0.0–34.5)

Abbreviations: ECOG PS, Eastern Cooperative Oncology Group performance status; LC, liver cirrhosis; HBV, hepatitis B virus; HCV, hepatitis C virus; AFP, α-fetoprotein; Non-major, sectional and segmental branch; Major, main or first branch; AJCC stage, American Joint Committee on Cancer stage; BCLC stage, Barcelona Clinic Liver Cancer stage; ALBI, albumin-bilirubin; Tx, treatment; AP, Adriamycin plus cisplatin; EQD2, equivalent dose in 2 Gy fractions (EQD2 = [(fraction dose + α/β)/(2 + α/β)] × total dose, α/β = 10); GyE, gray equivalent (GyE = proton physical dose [in gray] × relative biologic effectiveness [1.1]); _RRL_V_27GyE_, relative volume of the remaining residual liver receiving ≥ 27 GyE; _TL_V_27GyE_, relative volume of the total liver receiving ≥ 27 GyE; and D_2cc_, delivered radiation dose to the stomach, esophagus, duodenum, bowel, and spinal cord of 2 cc (^3^).

**Table 2:** Univariate analysis of pretreatment characteristics associated with freedom from local progression (FFLP), progression-free survival (PFS), and overall survival (OS).

			FFLP		PFS		OS	
Characteristics		N	5 Year (95% CI), %	*p* Value *	5 Year (95% CI), %	*p* Value *	5 Year (95% CI), %	*p* Value *
Sex	Male	35	93.4 (84.5–102.2)	0.738	37.5 (7.4–67.6)	0.412	65.8 (49.2–82.5)	0.360
	Female	11	90.0 (71.4–108.6)		63.6 (47.7–79.5)		80.8 (56.9–104.7)	
Age, years	<65	27	91.8 (80.8–102.8)	0.813	43.3 (24.3–62.3)	0.927	68.3 (49.7–86.9)	0.679
	≥65	19	93.8 (81.8–105.8)		46.3 (23.4–69.2)		72.4 (51.6–93.2)	
Etiology of LC	HBV	29	92.4 (82.2–102.6)	0.952	46.9 (28.3–65.5)	0.521	73.9 (56.8–91.0)	0.117
	Others	17	92.9 (79.4–106.4)		35.3 (8.6–62.0)		59.0 (32.3–85.7)	
Child–Pugh score	5	41	91.9 (83.1–100.7)	0.563	47.1 (31.2–63.0)	0.289	73.5 (59.0–88.0)	0.092
	6–7	5	100 (–) ^†^		0.0 (–) ^†^		30.0 (−16.8–76.8) ^†^	
AFP, ng/mL	<10	22	89.5 (75.8–103.2)	0.507	32.1 (10.9–53.3)	0.507	56.7 (32.0–81.4)	0.119
	≥10	24	95.7 (87.3–104.1)		53.6 (33.4–73.8)		79.2 (62.9–95.5)	
Tumor size, cm	<3	26	92.7 (82.7–102.5)	0.947	56.3 (37.7–74.9)	0.078	84.9 (71.2–98.6)	0.030
	≥3	20	93.3 (77.7–109.0)		23.3 (3.1–43.5)		45.8 (20.9–70.7)	
Tumor location	Hilar	19	87.4 (70.9–103.9)	0.261	36.1 (14.1–58.1)	0.333	57.4 (34.9–79.9)	0.155
	Non-hilar	27	96.0 (88.4–103.6)		48.3 (28.1–68.5)		76.7 (57.9–95.5)	
Vascular invasion	No/Non-major	41	94.7 (87.4–102.0)	0.022	46.0 (29.9–62.1)	0.043	75.1 (60.6–89.6)	0.011
	Major	5	66.7 (13.4–120.0)		20.0 (−15.1–55.1)		20.0 (−15.1–55.1)	
AJCC stage	I/II	40	94.7 (87.5–102.0)	0.037	47.2 (30.7–63.7)	0.007	77.0 (62.5–91.5)	0.001
	III/IV	6	75.0 (32.5–117.5)		16.7 (−13.1–46.5)		16.7 (−13.1–46.5)	
BCLC stage	0/A/B	39	94.5 (87.1–102.0)	0.110	46.5 (30.0–63.0)	0.053	76.7 (58.1–95.3)	0.004
	C	7	80.0 (44.9–115.1)		28.6 (−4.9–62.1)		28.6 (−4.9–62.1)	
ALBI grade	1	11	100 (–)	0.284	63.6 (35.2–92.0)	0.099	85.7 (59.8–111.6)	0.042
	2/3	35	89.9 (79.1–100.7)		35.4 (17.8–53.0)		57.8 (39.0–76.6)	
Total dose	≤90	7	83.3 (53.5–113.1)	0.203	14.3 (−11.6–40.2)	0.037	14.3 (−11.6–40.2)	0.004
(EQD2, GyE_10_)	>90	39	94.4 (86.8–102.0)		48.4 (31.7–65.1)		76.3 (61.4–91.2)	

Abbreviations: CI, confidence interval; all others are the same as in Table 1. * log-rank test. ^†^ 4 years.

**Table 3:** Multivariate analysis of pretreatment characteristics associated with freedom from local progression (FFLP), progression-free survival (PFS), and overall survival (OS).

		FFLP		PFS		OS	
Characteristics		Hazard Ratio (95% CI)	*p* Value *	Hazard Ratio (95% CI)	*p* Value *	Hazard Ratio (95% CI)	*p* Value *
AJCC stage	I/II	-	-	1.000	0.002	1.000	0.001
	III/IV	-		0.162 (0.051–0.511)		0.101 (0.027–0.384)	
ALBI grade	1	-	-	1.000	0.030	1.000	0.035
	2/3	-		0.282 (0.090–0.882)		0.098 (0.011–0.847)	

Abbreviations: same as in Table 1 and Table 2. * Cox proportional hazards model.

**Table 4:** Adverse events after proton beam radiotherapy according to albumin-bilirubin (ALBI) grade.

	All Patients (n = 46)				ALBI Grade		
CTCAE Grade	Grade 1, n (%)	Grade 2, n (%)	Grade 3, n (%)	Grade 4, n (%)	1, n (%)	2/3, n (%)	*p* Value *
Hematologic AEs	18 (39.1)	2 (4.3)	0 (0.0)	0 (0.0)	6 (54.5)	14 (40.0)	0.494
WBC increase	0 (0.0)	0 (0.0)	0 (0.0)	0 (0.0)			
WBC decrease	5 (10.9)	2 (4.3)	0 (0.0)	0 (0.0))			
PLT decrease	7 (15.2)	0 (0.0)	0 (0.0)	0 (0.0)			
ALT/AST increase	10 (21.7)	0 (0.0)	0 (0.0)	0 (0.0)			
Albumin decrease	1 (2.2)	0 (0.0)	0 (0.0)	0 (0.0)			
Bilirubin increase	1 (2.2)	0 (0.0)	0 (0.0)	0 (0.0)			
Non-hematologic AEs	16 (38.4)	2 (4.3)	0 (0.0)	0 (0.0)	4 (36.4)	14 (40.0)	1.000
Fever	0 (0.0)	0 (0.0)	0 (0.0)	0 (0.0)			
Pain	1 (2.2)	1 (2.2)	0 (0.0)	0 (0.0)			
Nausea	0 (0.0)	0 (0.0)	0 (0.0)	0 (0.0)			
Bleeding	0 (0.0)	0 (0.0)	0 (0.0)	0 (0.0)			
Dermatitis	11 (23.9)	2 (4.3)	0 (0.0)	0 (0.0)			
Radiation pneumonitis	7 (15.2)	0 (0.0)	0 (0.0)	0 (0.0)			
Upper gastrointestinal ulcer	2 (4.3)	0 (0.0)	0 (0.0)	0 (0.0)			

Abbreviations: CTCAE, Common Terminology Criteria for Adverse Events (Version 4.03); n, number of patients; WBC, white blood cell; PLT, platelet; ALT, alanine aminotransferase; AST, aspartate aminotransferase. * Fisher’s exact test, two-tail.

## Data Availability

The dataset used for this study is available upon request from the corresponding author.

## Supplementary Materials

The following supporting information can be downloaded at https://www.mdpi.com/article/10.3390/cancers14184445/s1, Figure S1: Definition of Target volumes and disease progressions; Figure S2: Freedom from Local progression (FFLP) (A), progression-free survival (PFS) (B), and overall survival (OS) (C) curves in patients with treatment-naïve hepatocellular carcinoma treated with proton beam therapy; Figure S3: Freedom from Local progression (FFLP) curves according to vascular invasion, AJCC stage, and EQD2 in all patients; Table S1: Subsequent treatment modalities for disease progression; Table S2: Change of Child–Pugh score and albumin-bilirubin (ALBI) grade after proton beam therapy.
