# Supplementary material for: Proton Beam Therapy for Treatment-Naïve Hepatocellular Carcinoma and Prognostic Significance of Albumin-Bilirubin (ALBI) Grade
Source: Cancers (Basel). 2022 Sep 13;14(18):4445. doi: 10.3390/cancers14184445 (PMC9497120; doi:10.3390/cancers14184445)
Supplement: Supplementary file 1 [file cancers-14-04445-s001.zip › cancers-1870256-supplementary.pdf]

Supplementary Materials

Supplementary Figures

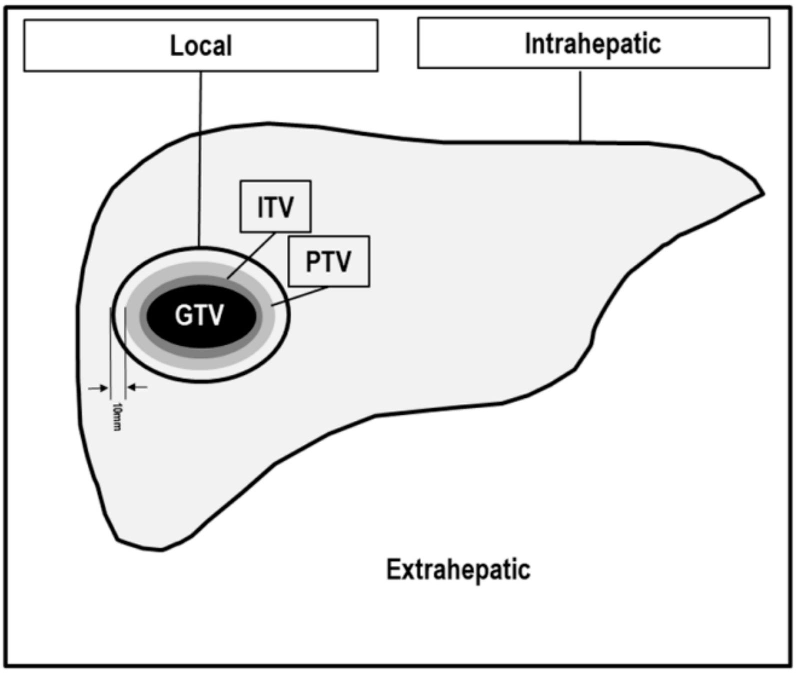

Supplementary Figure S1. Definition of Target volumes and disease progressions. Abbreviations: GTV, gross tumor volume; ITV, internal target volume (internal target volume); and planning target volume; Local, local progression; Intrahepatic, intrahepatic progression; and Extrahepatic, Extrahepatic progression.

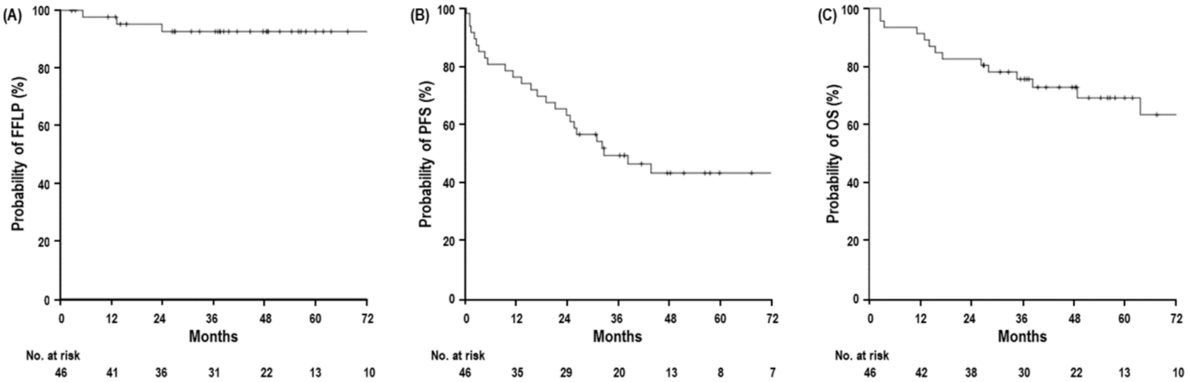

Supplementary Figure S2. Freedom from Local progression (FFLP) (A), progression-free survival (PFS) (B), and overall survival (OS) (C) curves in patients with treatment-naïve hepatocellular carcinoma treated with proton beam therapy.

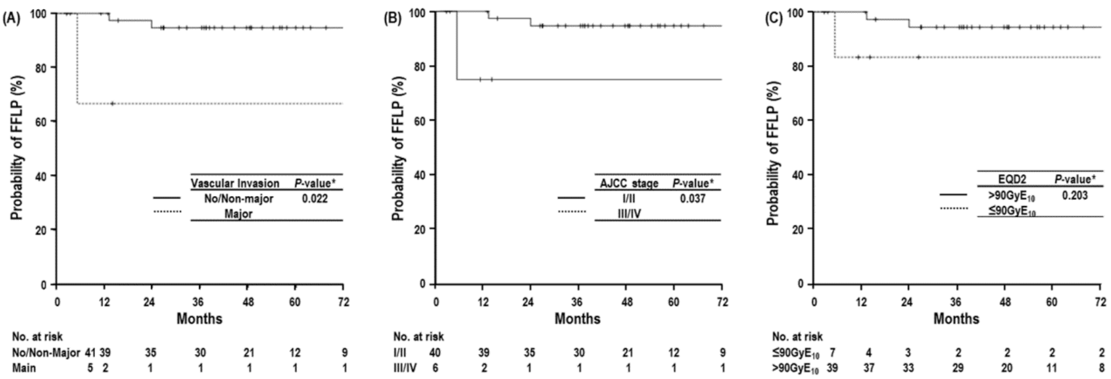

Supplementary Figure S3. Freedom from Local progression (FFLP) curves according to vascular invasion, AJCC stage, and EQD2 in all patients.

Abbreviations: Non-major, sectional and segmental branch; Major, main or first branch; AJCC stage, American Joint Committee on Cancer stage; EQD2, equivalent dose in 2 Gy fractions; and GyE, gray equivalent. * log-rank test.

Supplementary Table

Supplementary Table S1. Subsequent treatment modalities for disease progression

Details of subsequent Tx		Distribution, n (%)
Post-Tx to PBT lesion(s)	No	43 (83.5)
	Yes	3 (6.5)
	TACE	2 (4.3)
	SAT*	1 (2.2)
Post-Tx to non-PBT lesion(s)	No	23 (50.0)
	Yes	23 (50.0)
	TACE	8 (17.4)
	TACE ±RFA/PBT	2 (4.3)
	TACE ± RT + SAT*	2 (4.3)
	PBT	2 (4.3)
	RFA	1 (2.2)
	TARE + LT	1 (2.2)
	RFA+TACE+SAT*	1 (2.2)
	SAT*± RT	6 (2.2)

Abbreviations: n, number of patients; Tx, treatment; TACE, transarterial chemoembolization; RFA, radiofrequency ablation; PBT, proton beam therapy; TARE, transarterial radioembolization; LT, liver trasplantation; SAT, systemic anticancer therapy; and RT, radiotherapy.

*Sorafenib (n=7), lenvatinib (n=1), and doxorubicin plus cisplatin (n=1)

Supplementary Table S2. Change of Child-Pugh score and albumin-bilirubin (ALBI) grade after proton beam therapy

Change of Child–Pugh score	-1, n (%)	0, n (%)	+1, n (%)	+2, n (5)	*p value**
All patients	3 (6.5)	42 (91.3)	1 (2.2)	0 (0.0)	
ALBI grade					
1	0 (0.0)	11 (100)	0 (0.0)	0 (0.0)	0.669
2/3	3 (6.5)	31 (88.6)	1 (2.9)	0 (0.0)	
Tumor location					
Hilar	1 (5.3)	18 (94.7)	0 (0.0)	0 (0.0)	1.000
Non-hilar	2 (7.4)	24 (88.9)	1 (3.7)	0 (0.0)	
**Change of ALBI grade**	**-1, n (%)**	**0, n (%)**	**+1, n (%)**	**+2, n (5)**	***p value****
All patients	0 (0.0)	42 (91.3)	4 (8.7)	0 (0.0)	
Tumor location					
Hilar	0 (0.0)	17 (89.5)	2 (10.5)	0 (0.0)	1.000
Non-hilar	0 (0.0)	25 (92.6)	2 (7.4)	0 (0.0)	

*Fisher's exact test, two-tail.
